# The use, adherence, and evaluation of interactive text-messaging among women admitted to prevention of mother-to-child transmission of HIV care in Kenya (WelTel PMTCT)

**DOI:** 10.1186/s12884-023-06194-0

**Published:** 2024-01-03

**Authors:** Björn Nordberg, Eunice Kaguiri, Katrine J. Chamorro de Angeles, Erin E. Gabriel, Mia Liisa van der Kop, Winfred Mwangi, Richard T. Lester, Edwin Were, Anna Mia Ekström, Susanne Rautiainen

**Affiliations:** 1 Department of Global Public Health, Karolinska Institutet, Stockholm, Sweden; 2 Department of Infectious Diseases, Helsingborg Hospital, Helsingborg, Sweden; 3 Partners in Prevention, Moi University, Eldoret, Kenya; 4 Department of Medical Epidemiology and Biostatistics, Karolinska Institutet, Stockholm, Sweden; 5 Directorate of Reproductive Health, Moi Teaching and Referral Hospital, Eldoret, Kenya; 6 Department of Medicine, Division of Infectious Diseases, University of British Columbia, Vancouver, Canada; 7 Department of Reproductive Health, Moi University, Eldoret, Kenya; 8 Department of Infectious Diseases, South General Hospital, Stockholm, Sweden; 9 Department of Medicine, Clinical Epidemiology Division, Karolinska Institutet, Stockholm, Sweden

**Keywords:** HIV, Pregnant women, PMTCT, WHO Option B +, Text-messaging, MHealth, Kenya

## Abstract

**Background:**

To improve future mobile health (mHealth) interventions in resource-limited settings, knowledge of participants’ adherence to interactive interventions is needed, but previous studies are limited. We aimed to investigate how women in prevention of mother-to-child transmission of HIV (PMTCT) care in Kenya used, adhered to, and evaluated an interactive text-messaging intervention.

**Methods:**

We conducted a cohort study nested within the WelTel PMTCT trial among 299 pregnant women living with HIV aged ≥ 18 years. They received weekly text messages from their first antenatal care visit until 24 months postpartum asking “How are you?”. They were instructed to text within 48 h stating that they were “okay” or had a “problem”. Healthcare workers phoned non-responders and problem-responders to manage any issue. We used multivariable-adjusted logistic and negative binomial regression to estimate adjusted odds ratios (aORs), rate ratios (aRRs) and 95% confidence intervals (CIs) to assess associations between baseline characteristics and text responses. Perceptions of the intervention were evaluated through interviewer-administered follow-up questionnaires at 24 months postpartum.

**Results:**

The 299 participants sent 15,183 (48%) okay-responses and 438 (1%) problem-responses. There were 16,017 (51%) instances of non-response. The proportion of non-responses increased with time and exceeded 50% around 14 months from enrolment. Most reported problems were health related (84%). Having secondary education was associated with reporting a problem (aOR:1.88; 95%CI: 1.08–3.27) compared to having primary education or less. Younger age (18–24 years) was associated with responding to < 50% of messages (aOR:2.20; 95%CI: 1.03–4.72), compared to being 35–44 years. Women with higher than secondary education were less likely (aOR:0.28; 95%CI: 0.13–0.64), to respond to < 50% of messages compared to women with primary education or less. Women who had disclosed their HIV status had a lower rate of non-response (aRR:0.77; 95%CI: 0.60–0.97). In interviews with 176 women, 167 (95%) agreed or strongly agreed that the intervention had been helpful, mainly by improving access to and communication with their healthcare providers (43%).

**Conclusion:**

In this observational study, women of younger age, lower education, and who had not disclosed their HIV status were less likely to adhere to interactive text-messaging. The majority of those still enrolled at the end of the intervention reported that text-messaging had been helpful, mainly by improving access to healthcare providers. Future mHealth interventions aiming to improve PMTCT care need to be targeted to attract the attention of women with lower education and younger age.

**Supplementary Information:**

The online version contains supplementary material available at 10.1186/s12884-023-06194-0.

## Background

There has been an explosive expansion in mobile phone ownership in Sub-Saharan Africa (SSA). By the end of 2021, 515 million people (46% of the SSA population) had a mobile phone subscription [1]. With continued growth to an estimated 100 million new mobile phone subscribers in 2025, opportunities to use mobile health (mHealth) technologies to improve health outcomes in low- and middle-income countries [1], including women’s and maternal health outcomes, are expanding. A 2019 systematic review including 30 studies from low- and middle-income countries, concluded that mHealth interventions can improve outcomes of maternal health, antenatal care, postnatal care, HIV prevention, vaccination, and adherence to treatment [2]. In addition, text-messaging interventions have been reported to improve care of cardiovascular disease, diabetes mellitus, tuberculosis, and anaemia in low- and middle-income countries [2].

Interactive text-messaging and phone call support can be used for patient tracking, counselling, and education. Studies investigating the impact of text-messaging in HIV care including the prevention of mother-to-child transmission of HIV (PMTCT) care have reported mixed results on adherence to antiretroviral therapy (ART) [3–5] or retention in care [3, 5–8]. To optimize future mHealth interventions aiming to improve outcomes in healthcare including HIV and PMTCT care, it is important to investigate how participants use and adhere to interactive text-messaging interventions. Adherence to text-messaging interventions to improve outcomes of HIV care and to collect HIV-related data have, however, been observed to vary widely depending on topic, population, and setting [9].

WelTel is an intervention involving weekly text-messaging with follow-up phone calls, if required, to enable prompt assistance and counselling. The aim is to increase patients’ engagement in HIV care through improved communication with healthcare workers [10]. The WelTel intervention has previously been investigated in two randomised controlled trials (RCTs) and was found to improve adherence to ART and HIV viral suppression [11], but not retention in HIV care [12]. The response rate to interactive text-messaging in previous WelTel trials varied between 55% [12] and 68% [11]. In the WelTel Kenya1 trial, reporting a problem was associated with younger age and rural residence, and non-adherence to interactive text-messaging was associated with lower education and study site [13]. A trial of another interactive text-messaging intervention reported a 63% response rate during 20 weeks of follow-up and an improved HIV viral load count in the intervention group [14]. Moreover, one pilot study from South Africa of women in PMTCT care reported that more than 50% of women used text-messaging or phone calls to initiate a contact with a healthcare counsellor [15].

Pregnant and postpartum women living with HIV face unique challenges compared to other populations due to problems related to pregnancy, childbirth, and taking care of a new-born infant, factors which can increase barriers to retention in care and may require women to receive additional support [16, 17]. The objective of this study was to investigate how women in PMTCT care in Kenya used, adhered to, and evaluated a weekly interactive text-messaging intervention.

## Methods

### Study design, setting and population

This is a longitudinal cohort study nested within the WelTel PMTCT trial of 299 pregnant and postpartum women allocated to the text message intervention group. WelTel PMTCT was a randomised, two-armed, parallel-group trial carried out at six clinics that offered antenatal, postnatal and PMTCT care at the Moi Teaching and Referral Hospital (MTRH), Kitale County Referral Hospital, Uasin Gishu District Hospital (UGDH), Huruma Sub-County Hospital, Chulaimbo Sub-County Hospital, and Matayos Sub-County Hospital in western Kenya [18]. Pregnant women 18 years or older, living with HIV or newly diagnosed with HIV who presented for their first antenatal care visit in the current pregnancy, were enrolled between June 2015 and July 2016. Participants needed to have access to a mobile phone, be able to respond or have someone to respond to text messages, and plan on being a resident of the clinic catchment area up to 24 months postpartum. The World Health Organization (WHO) PMTCT Option B + guidelines had been adopted at the clinics, and outreach tracing of women who defaulted on follow-up visits had been implemented as part of routine PMTCT care. We obtained ethical approval from the Institutional Research and Ethics Committee at Moi University, Kenya (FAN: IREC 1292) and the Regional Ethics Committee, Stockholm, Sweden (2018/742–31/1). Written informed consent was provided by all participants before trial enrolment. All trial procedures were performed in accordance with relevant guidelines and regulations.

### The WelTel intervention

At enrolment, the participants’ mobile phone numbers were registered in the WelTel platform, and the text message intervention started the following week. Participants received an automatic, weekly text message every Monday morning asking “Mambo?” (Swahili for “How are you?”) in addition to routine PMTCT care. The participants were instructed to respond (in Swahili or English) within 48 h either that they were “okay” (e.g., “sawa” in Swahili) or that they had a “problem” (e.g., “shida” in Swahili). When participants responded with a problem, their texts were automatically forwarded to a mobile phone at the participants’ clinic. Within 48 h, a healthcare worker made a follow-up phone call to the participants who had reported a problem to provide counselling and assistance. In addition, a list of non-responders was delivered every week to the local clinic. The healthcare workers called the non-responders to determine the reason for not responding and whether they needed assistance.

The weekly text messages continued until 24 months postpartum. The intervention was stopped in the case of miscarriage, stillbirth, infant death, maternal death, or participant withdrawal from the intervention or study follow-up, as soon as this was reported to the central study coordinator. The participants’ time in the intervention also varied depending on gestational age at enrolment. At enrolment, participants were informed that the text-messaging intervention did not replace routine clinic visits and services, and that they should contact the clinic using usual methods in case of emergencies.

### Baseline characteristics and outcome measures

The WelTel platform automatically recorded participants’ responses and non-responses using the following categories: okay, problem, or non-response. Healthcare workers categorized problems reported by participants during the follow-up phone calls into predetermined categories and then recorded these issues in a paper log. The problems were classified as health related, social or domestic, economic or financial, psychological, or in need of information. Detailed information about a limited number of specified problems (*n* = 43) was obtained from the paper logs. Information about detailed problems was also obtained from post-intervention focus group evaluations with healthcare workers who had done the follow-up phone calls to the participants (unpublished work). Healthcare workers also entered the participants’ open-ended reasons for not responding to text messages in a paper log (categorized after data collection). At study enrolment, women responded to a structured questionnaire administered face-to-face by a research assistant about socio-demographic and HIV-related characteristics. The questions included age, educational level, marital- and cohabitation status, time since HIV diagnosis, HIV status disclosure, travel time to the clinic, and which phone they would use in the study.

At 24 months postpartum, a structured follow-up questionnaire was administered face-to-face by a research assistant to 176 (58.9%) out of the 299 participants enrolled. The participants were asked whether the weekly text messages were helpful (strongly disagree, disagree, neither disagree or agree, agree, strongly agree), if they wanted the text-messaging program to continue (yes, no, do not know), and open-ended questions about the greatest perceived benefits and barriers of the weekly text message communication, which were categorized after data collection.

### Statistical analysis

Frequencies and proportions were used to summarize baseline characteristics of the study population, outcomes of text-messaging, outcomes of follow-up phone calls, and outcomes of the follow-up interviews. Where appropriate, means and standard deviations (SDs), and medians and interquartile ranges (IQRs) were used. The proportion of text messages indicating a problem sent during pregnancy and during the postpartum period was calculated for those with an indicated date of delivery (*n* = 249). Multivariable-adjusted logistic regression was used to estimate adjusted odds ratios (aORs) and 95% confidence intervals (CIs) for the association between baseline characteristics and (1) reporting a problem at least once and (2) responding to < 50% of the text messages received.

Our count data for non-responses to text messages was overdispersed. To examine the association between the number of non-responses and baseline characteristics we used multivariable-adjusted negative binomial regression to estimate adjusted rate ratios (aRRs) and 95% CIs. Based on previous literature of factors associated with reporting problems in text-messaging interventions or retention in PMTCT care, covariates included in the analyses were determined a priori before any statistical analyses were carried out. Odds ratios and rate ratios were adjusted for the following factors: age [13, 19, 20]; educational level [19, 21]; being married or living with a partner [22]; time since HIV diagnosis [23]; HIV status disclosure [19, 24]; travel time to clinic [19]; phone used in the study [13]; and clinic of enrolment [25]. Odds ratios were also adjusted for the total number of text messages received in the study. Rate ratios in the negative binomial regression analysis also included an offset for the log of the total number of text messages received. The Matayos and Chulaimbo clinics were combined in regression analyses due to few participants at the Matayos clinic. The clinics were similar in terms of demography, geography, and HIV prevalence. StataCorp. 2017. *Stata Statistical Software: Release 15*. College Station, TX: StataCorp LLC was used to perform data analyses.

## Results

Between June 29th, 2015, and December 31st, 2018, participants received 31,640 text messages until the last participant had reached 24 months postpartum. Two text messages were sent to a participant after withdrawal from study follow-up and were therefore excluded, leaving 31,638 text messages in the analyses. The participants received the text messages for a median of 27 (IQR 25–29) months. Text messages were stopped before 24 months postpartum due to miscarriage (*n* = 22), stillbirth (*n* = 2), death of the infant (*n* = 5), death of the participant (*n* = 1), withdrawal from receiving text messages (*n* = 16), and withdrawal from study follow-up (*n* = 1). In addition, 13 participants received the intervention shorter than intended for technical or other reasons. The population’s characteristics at study enrolment are presented in Table 1. Half the population (*n* = 147, 49.2%) had primary schooling or less, and most (*n* = 244, 81.6%) were married or living with a partner. More than two-thirds (*n* = 206, 68.9%) were diagnosed with HIV at least six months before trial enrolment, and most study participants (*n* = 226, 75.6%) had disclosed their HIV status. Almost three-quarters (*n* = 214, 71.6%) of participants had less than one hour travel time to their clinic.

**Table 1:** Baseline characteristics (*n* = 299)

Characteristic	n (%)
**Age (years)**	
18–24	67 (22.4)
25–29	79 (26.4)
30–34	83 (27.8)
35–44	70 (23.4)
	mean = 29.6 SD = 5.9
**Education**	
Primary schooling or less	147 (49.2)
Secondary schooling	105 (35.1)
Higher education	47 (15.7)
**Married or living with a partner**	
Yes	244 (81.6)
No	55 (18.4)
**Time since HIV diagnosis**	
< 6 months	93 (31.1)
≥ 6 months	206 (68.9)
**HIV status disclosure**	
Yes	226 (75.6)
No	73 (24.4)
**Travel time to clinic**	
< 1 h	214 (71.6)
≥ 1 h	85 (28.4)
**Phone used in study**	
Own phone	271 (90.6)
Another phone	28 (9.4)
**Clinic of enrolment**	
Chulaimbo	40 (13.4)
Huruma	23 (7.7)
Kitale	102 (34.1)
Matayos	3 (1.0)
MTRH	93 (31.1)
UGDH	38 (12.7)

*SD* Standard deviation, *MTRH* Moi Teaching and Referral Hospital, *UGDH* Uasin Gishu District Hospital

### Text message outcomes

**Fig. 1:**
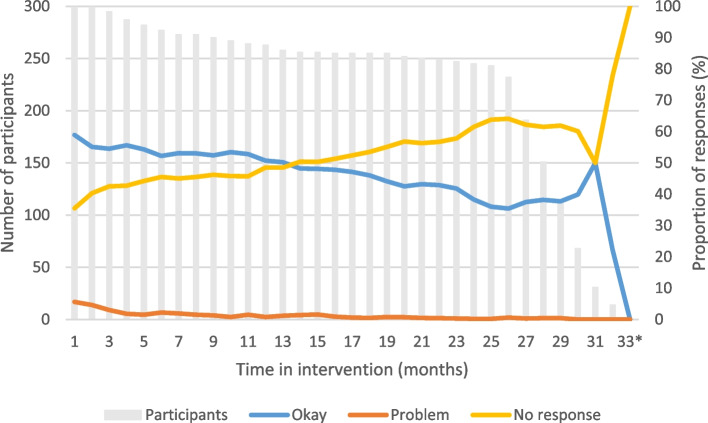
Number of participants receiving weekly text messages and the proportion of participants’ text message responses (*n* = 299). *One participant received the intervention for 33 months. The participants’ time in the intervention differed depending on gestational age at enrolment

In total, 15,183 (48.0%) okay responses, 438 (1.4%) problem responses, and 16,017 (50.6%) instances of non-response were included in the analysis. Of a median of 118 (IQR 109–126) text messages sent to each participant, the women sent a median of 54 (IQR 10–83) okay responses and a median of 0 (IQR 0–2) problem responses. A median of 45 (IQR 20–83) non-responses were registered for each participant. Figure 1 demonstrates how the proportion of problem responses decreased from 5.6% during the participants’ first month in the intervention to 0.4% during the participants’ 28th month in the intervention (median time of follow-up). Okay responses decreased from 58.9% to 38.2%, and non-responses increased from 35.5% to 61.5% from the first month to the 28th month of the intervention. The proportion of non-responses exceeded 50% around 14 months from enrolment and continued to increase thereafter.

### Problems reported

Out of the 299 participants, 147 (49.2%) sent one or more text responses indicating a problem. During the follow-up phone calls, healthcare workers categorized the reported problems. In nine of these instances, the reported problems were classified in more than one category. This resulted in a total of 447 problems that were indicated by healthcare workers. However, three (0.7%) of the 447 problems were never categorized. The most reported problems were health related (83.9%), followed by social or domestic (5.4%), economic or financial (4.3%), psychological (3.6%), and need of information (2.2%). More than half of the problem responses (53.2%) were sent by participants during their first six months in the study. Among the 249 women with an indicated date of delivery, the proportion of problem responses sent during pregnancy was 129 (3.3%) out of 3,969 text messages received. The proportion of problem responses sent postpartum was 257 (1.1%) out of 24,410 text messages received. The proportion of problems reported the last month before delivery was 2.6% (*n* = 27), the first month postpartum 4.4% (*n* = 44) and the second month postpartum 2.2% (*n* = 23).

In 43 (9.6%) of the 447 problems reported at follow-up calls, more detailed information about the specific problem was collected. Among health-related problems (*n* = 34), problems related to women’s health were more reported (*n* = 25, 73.5%) than problems related to infants’ health (*n* = 9, 26.5%). Examples of health-related problems included feeling sick in general, having malaria, ART side-effects, miscarriage, abdominal pain, problems related to delivery, nosebleed, or the infant having diarrhoea or stomach problems. Social or domestic problems were related to relationship issues with the husband or partner, or with family or friends. Examples of economic or financial problems were eviction due to non-payment of rent, difficulty managing the costs associated with attending the clinic or their infant’s medical condition. Psychological problems included stress, depression, grief, trauma, or stigma. Need of information was related to family planning, ART, or how to access ART if the medication was lost.

### Non-responses to text messages

The participants’ reasons for not responding to text messages are found in Table 2. The most common reasons for not responding were that the participants were busy or forgot to respond (12.1%) or had a phone-related problem (9.1%) (i.e., the phone had stopped working, network problems, battery problems, etc.). In 9.3% of non-responses the mobile phone was turned off either when the follow-up call was made so that the participant could not be reached, or when the text message was sent and during the 48-h window period of responding to the message. The reason for not responding to a text message could not be determined in most cases because the participant did not answer the healthcare workers’ follow-up phone call (53.5%); someone else answered the call (1.0%); or the phone number of the participant was incorrect (1.0%). Also, there were instances when a follow-up call was not made (1.6%) or where data were missing (0.4%). During follow-up, 130 (43.5%) of the 299 participants responded to < 50% of the text messages, and 12 participants who received the intervention for a median of 27 (IQR 26–29) months did not respond to any text message. Only one participant who received the full intervention until 24 months postpartum responded to all the text messages.

**Table 2:** Reasons for not responding to text messages (*n* = 16,017 non-responses)

Outcome	n (%)
Busy or forgot	1,937 (12.1)
Phone-related problem	1,459 (9.1)
Did not have access to the phone	778 (4.9)
Claims that a text message response was sent	689 (4.3)
Phone turned off	1,486 (9.3)
No answer to follow-up phone call	8,562 (53.5)
No follow-up phone call to participant	263 (1.6)
Wrong number	164 (1.0)
Someone else answered the follow-up call	154 (1.0)
Other reason	416 (2.6)
No reason	38 (0.2)
Reason missing	71 (0.4)

### Multivariable-adjusted analyses of reporting problems and non-response

The logistic regression analysis of the association between baseline characteristics and reporting a problem at least once is reported in Table 3. Having secondary schooling as the highest level of education compared to primary schooling or less was associated with reporting at least one problem (aOR: 1.88; 95% CI: 1.08–3.27), the corresponding aOR for higher than secondary education was 1.58; (95% CI: 0.77–3.22). When education level was dichotomized (secondary schooling and higher education combined vs. primary schooling or less), leaving all other covariates unchanged, the association was statistically significant (aOR: 1.78; 95% CI: 1.07–2.96) (Additional file 1). Additionally, clinic of enrolment was significantly associated with reporting a problem at least once at the Kitale clinic (aOR: 2.39; 95% CI: 1.29–4.44) compared to the MTRH (reference) clinic (Table 3).

**Table 3:** Association of baseline characteristics (*n* = 299) and reporting a problem or responding to < 50% of text messages

Characteristic	Responded with a problem aOR (95% CI)	*p*-value	Responded to < 50% of the text messages aOR (95% CI)	*p*-value
**Age (years)**				
18–24	1.26 (0.59—2.66)	0.55	2.20 (1.03—4.72)	0.04
25–29	1.09 (0.54—2.19)	0.81	1.31 (0.64—2.65)	0.46
30–34	1.66 (0.83—3.30)	0.15	1.05 (0.52—2.12)	0.89
35–44	Ref		Ref	
**Education**				
Primary schooling or less	Ref		Ref	
Secondary schooling	1.88 (1.08—3.27)	0.03	0.74 (0.43—1.28)	0.28
Higher education	1.58 (0.77—3.22)	0.21	0.28 (0.13—0.64)	< 0.01
**Married or living with a partner**				
Yes	1.06 (0.56—2.01)	0.85	1.03 (0.54—1.97)	0.93
No	Ref		Ref	
**Time since HIV diagnosis**				
< 6 months	0.65 (0.32—1.31)	0.23	0.94 (0.46—1.91)	0.86
≥ 6 months	Ref		Ref	
**Disclosure of HIV status**				
Yes	1.01 (0.50—2.05)	0.97	0.52 (0.25—1.06)	0.07
No	Ref		Ref	
**Travel time to clinic**				
< 1 h	Ref		Ref	
≥ 1 h	1.13 (0.64—2.00)	0.68	1.36 (0.77—2.43)	0.29
**Phone used in study**				
Own phone	1.54 (0.65—3.67)	0.33	0.84 (0.36—1.96)	0.69
Another phone	Ref			
**Clinic of enrolment**				
MTRH	Ref		Ref	
Chulaimbo + Matayos^a^	1.35 (0.62—2.94)	0.46	1.24 (0.56—2.74)	0.59
Kitale	2.39 (1.29—4.44)	0.01	0.85 (0.45—1.58)	0.61
Huruma	0.54 (0.18—1.60)	0.27	0.73 (0.27—2.01)	0.55
UGDH	1.55 (0.67—3.56)	0.31	0.55 (0.23—1.32)	0.18

*aOR* adjusted Odds Ratio, *CI* Confidence Interval, *MTRH* Moi Teaching and Referral Hospital, *UGDH* Uasin Gishu District Hospital

The models were also adjusted for the number of text messages received in the study

^a^The Chulaimbo and Matayos clinics were combined in multivariate analyses due to few participants at the Matayos clinic. The clinics were similar in terms of demography, geography, and HIV prevalence

In the logistic regression analysis of the association between baseline characteristics and responding to < 50% of text messages (Table 3), women 18–24 years old (aOR: 2.20; 95% CI: 1.03–4.72) had higher odds of responding to < 50% of text messages compared to women 35–44 years old. Having higher than secondary education compared to primary education or lower was associated with lower odds (aOR: 0.28; 95% CI: 0.13–0.64) of responding to < 50% of text messages.

In the negative binomial regression analysis (Additional file 2), women 18–24 years old had a borderline significant higher rate (aRR: 1.25; 95% CI: 0.99–1.59) of non-responses compared to women 35–44 years old. Having higher than secondary education was associated with a lower rate (aRR: 0.65; 95% CI: 0.51–0.82) of non-response to text messages compared to primary education or lower. In addition, having disclosed HIV status at study enrolment was associated with a lower rate (aRR: 0.77; 95% CI: 0.60–0.97) of non-response to text messages.

### Women’s evaluation of the intervention

At 24 months postpartum, 176 (58.9%) out of 299 participants responded to the interviewer-administered follow-up questionnaire. Of the 176 responders, 93 (52.8%) strongly agreed and 74 (42.0%) agreed that the weekly text messages had been helpful, whereas 4 (2.3%) neither disagreed nor agreed and 5 (2.8%) participants strongly disagreed. In addition, 129 (73.3%) replied that they would like the text message program to continue, whereas 27 (15.3%) did not want it to continue, and 20 (11.4%) were unsure. Participants felt that the greatest benefits of receiving the weekly text messages were improved communication and easy access to the healthcare worker (76/176, 43.2%). Other benefits included feeling that the healthcare workers cared for them (33/176, 18.8%), and that the text messages reminded them to attend clinic appointments (22/176, 12.5%). The greatest barriers to receiving and responding to the text messages were lack of phone access (27/176, 15.3%), lack of battery power (21/176, 11.9%), other phone-related problems (18/176, 10.2%), or being busy or forgetting to respond (20/176, 11.4%). Many participants (70/176, 39.8%) responded that there were no barriers to receiving and responding to text messages.

## Discussion

In this study of women in PMTCT care who were randomised to receive an interactive text-messaging intervention, we observed that the adherence to responding to text messages gradually decreased over time, reaching below 50% halfway through the intervention. Women below 25 years of age, with lower educational level and those who had not disclosed their HIV status had lower adherence. Educational level and study site were associated with using the intervention to report a problem. The most common reasons for non-adherence were being busy or forgetting to respond. Half of the women used the intervention to report at least one problem during the follow-up, and problems related to the woman’s or the infant’s health were most common. Among participants who were interviewed at the end of the intervention, a majority found the intervention helpful, and wanted it to continue.

Interactive text-messaging interventions have been used in RCTs with varying impact on outcomes in PMTCT care including early infant HIV testing and retention in care [5–8]. To evaluate the participants’ responses and adherence to interactive text-messaging interventions is important when interpreting trial results, and to be able to design more targeted and improved future interventions. However, this is to the best of our knowledge the first study to investigate the use and adherence to interactive text-messaging among women enrolled in PMTCT care. The proportion of responses to text messages, including problem responses, was lower in our study compared to previous intervention trials among men and women in general HIV care in Kenya [11, 12] and Nigeria [14]. One possible explanation may be the longer (more than two-year) duration of the intervention used in our trial, which may indicate text-messaging fatigue. Text messages that are interactive and sent less frequently than daily have been observed to reduce habituation and text-messaging fatigue among men and women living with HIV [4]. However, despite these elements in our intervention, our results suggest that two years may be too long to maintain participants’ engagement to stay adherent to interactive text-messaging. It is also possible that fatigue to respond to text messages may develop when the same message is sent every week, which was also observed in a previous trial of the WelTel intervention [11, 13]. Consistent with our results, a study among adolescents in the United States (US) observed a decreasing response rate after four months of an interactive text-messaging intervention [26]. The US study also reported that dynamic messages (quizzes and questions) generated more responses as compared to static messages [26].

The type and pattern of text messages sent in interventions may also be of importance to improve PMTCT care in SSA. Two qualitative studies from Kenya reported that women in PMTCT care preferred direct appointment reminders sent to them infrequently rather than repeated weekly messages [27], as well as educational and encouraging messages to improve emotional support [28]. In line with this, a 2014 meta-analysis of RCTs reported that interventions with personalized message content had a larger effect on adherence to ART than interventions without personalized messages among men and women living with HIV [4]. In contrast, a 2021 RCT from Kenya of 824 women in PMTCT care reported no effect on either ART adherence or two-year retention in care [5] of an intervention with tailored text messages that were timed along the pregnancy and postpartum continuum [29]. Moreover, a 2022 cluster-RCT from Kenya of 1,331 women in PMTCT care reported no effect on either ART adherence or one-year retention in care of tailored, weekly text messages [30].

Increasing information flow due to the rapid increase in mobile phone use, global mobile internet connectivity [31] and use of social media [32], may present a challenge to attract the attention of women in PMTCT care to remain adherent to mHealth interventions. This may also contribute to fatigue and unwillingness to respond. Women in our study reported that being busy or forgetting was the most common reason for not responding to text messages. Attracting the attention of certain groups of women may be particularly challenging. In our study, we observed that younger women, women with lower education, and women who had not disclosed their HIV status at enrolment were the least adherent to responding to text messages. These findings are consistent with the WelTel Kenya1 trial where educational level was associated with non-adherence to the intervention [13]. Similarly, younger age [19, 20], lower educational level [19, 21], and non-disclosure of HIV status [19, 24] are factors that have been associated with poor retention in PMTCT care. Our results indicate that further efforts and possibly other interventions may be needed to reach women in these groups to improve outcomes related to their care. Our findings also suggest that the duration and timing of mHealth interventions are important factors to consider in similar settings, as loss of phone access and user fatigue are barriers that need to be addressed. Additionally, women’s support needs may vary along the PMTCT cascade.

One of the main purposes of the WelTel intervention is to allow for patients to report problems to get in contact with their healthcare workers to get assistance and counselling. In our study, however, participants made limited use of the intervention to report a problem. Over a median of 27 months, half the women used the service to report a problem at least once. We also observed that participants used the service to report a problem most frequently during the first months after enrolment and during the first month postpartum. We observed that less than 10% of the problems were of social, domestic, or psychological nature, and we have previously reported that a low proportion of the WelTel PMTCT trial participants reported social and emotional barriers related to participation in PMTCT care at trial enrolment [33]. Thus, women may have experienced few social, domestic, or psychological problems, but it is also possible that the participants did not report those problems and instead considered the intervention more useful to get assistance of problems related to their physical health. Our results suggest that women with a lower educational level may be less likely to use text-messaging to report a problem. The association between educational level and sending a text message indicating a problem may be due to various reasons. A systematic review of 30 mHealth intervention studies from low- and middle-income countries reported that limited reading and writing skills to use a mobile phone were barriers to text-messaging interventions [2]. The WelTel intervention was, however, easy to use and educational level may also be associated with mobile phone usage patterns, or awareness of health issues associated with pregnancy and HIV. In contrast to our results, younger people living with HIV in Kenya have previously been observed to be more likely to report problems in a text-messaging intervention [13]. The inconsistency may be explained by narrower age range in our study, differing characteristics of the study populations, or a generally increased mobile phone use [34] among young and old people in Kenya.

Although the WelTel PMTCT trial reported no effect on early infant HIV testing [35] or 18-month retention in PMTCT care [36] for mother-infant-pairs, it allows for improved communication and support for participants [10]. Most participants who responded to the interviewer-administered questionnaire at the end of follow-up wanted the intervention to continue. The greatest perceived benefit of the intervention was that it enabled access to and contact with healthcare workers. In qualitative in-depth interviews, women enrolled in the WelTel PMTCT trial have reported that they felt safe having the option to text “problem” 24 h per day, 7 days per week, which was considered to be an important feature of the intervention [37]. Another benefit was that participants felt that the healthcare workers cared for them, which also is in line with qualitative assessments from our trial [37] and a study of the same intervention among people in general HIV care [13]. In addition, the WelTel intervention has previously been observed to improve ART adherence [11], HIV viral suppression [11], self-perceived health-related quality of life [12], and study participants in previous WelTel studies have reported that the text-messaging reminded them to take ART and to attend clinic appointments [13].

A key strength of this study is the long follow-up time and long duration of the text-messaging intervention, which covered the full PMTCT period from the first visit to 24 months postpartum. A high participation rate and a multicentre design that included sites from urban and rural areas across western Kenya contributed to the external validity of our results. The study also had several limitations. According to qualitative data collected in our trial, there were extensive informal non-intervention phone communication between some healthcare workers and participants alongside the intervention [37]. This may have impacted participants' response to text messages and their use of the intervention for assistance with potential problems. In addition, we were not able to measure whether participants opened or read the text messages. However, due to the nature of the intervention, participants were instructed to respond within 48 h and follow-up calls were made to those who did not respond. Despite this potential limitation in measuring message interaction, our data do not indicate issues with text message delivery except among the 13 participants who received a shorter duration of the intervention than intended. At the end of follow-up, 210 of 299 (70%) participants were retained in PMTCT care, which limited number of participants who we were able to interview to evaluate the intervention at 24 months postpartum. This dropout was due to miscarriages, stillbirths, death of the infant, death of the participant, transfer to another clinic, and lost to follow-up, and it may have contributed to a selection bias which could have influenced the 24-months postpartum interview results. The face-to-face collection of questionnaire data at baseline and 24 months postpartum may have contributed to social desirability bias which may have influenced these results due to a potential desire among the participants to portray themselves, and their perception of the intervention, in a way that they considered to be socially favourable.

## Conclusion

In this observational study of pregnant and postpartum women living with HIV in western Kenya, who were randomised to a text-messaging intervention, women of younger age, lower education, and who had not disclosed their HIV status were less likely to adhere to interactive text-messaging. The proportion of non-responses increased with time and exceeded 50% around 14 months from enrolment. The majority of those still enrolled at the end of the intervention reported that text-messaging had been helpful, mainly by improving access to healthcare providers. Future mHealth interventions aiming to improve outcomes in PMTCT care need to be targeted to attract the attention of women with lower education and younger age.

### Supplementary Information

**Additional file 1.** Logistic regression of reporting a problem and association with participants’ baseline characteristics, education variable dichotomized (*n*=299).

**Additional file 2.** Negative binomial regression of non-responses to weekly text messages and association with participants’ baseline characteristics (*n*=299).

## Data Availability

The datasets used and/or analysed during the current study are available from the corresponding author on reasonable request.
