# Supplementary material for: The use, adherence, and evaluation of interactive text-messaging among women admitted to prevention of mother-to-child transmission of HIV care in Kenya (WelTel PMTCT)
Source: BMC Pregnancy Childbirth. 2024 Jan 3;24:25. doi: 10.1186/s12884-023-06194-0 (PMC10763426; doi:10.1186/s12884-023-06194-0)
Supplement: Supplementary file 1 — Additional file 1. Logistic regression of reporting a problem and association with participants’ baseline characteristics, education variable dichotomized (n=299). [file 12884_2023_6194_MOESM1_ESM.docx]

**Additional file 1.** Logistic regression of reporting a problem and association with participants’ baseline characteristics, education variable dichotomized (n=299).

| Characteristic of participant at study enrolment. | Responded with a problem  aOR (95% CI) | p-  value |
| --- | --- | --- |
| **Age (years)**  18-24  25-29  30-34  35-44 | 1.26 (0.60 - 2.66)  1.09 (0.54 - 2.18)  1.65 (0.83 - 3.30)  Ref. | 0.55  0.81  0.15 |
| **Education**  Primary schooling or less  Secondary schooling or higher | Ref.  1.78 (1.07 - 2.96) | 0.03 |
| **Married or living with a partner**  Yes  No | 1.07 (0.57 - 2.02)  Ref. | 0.84 |
| **Time since HIV diagnosis**  < 6 months  ≥ 6 months | 0.65 (0.32 - 1.31)  Ref. | 0.23 |
| **Disclosure of HIV status:**  Yes  No | 1.02 (0.50 - 2.05)  Ref. | 0.96 |
| **Travel time to clinic**  < 1 hour  ≥ 1 hour | Ref.  1.12 (0.64 - 1.99) | 0.69 |
| **Phone used in study**  Own phone  Another phone | 1.55 (0.65 - 3.68)  Ref. | 0.33 |
| **Clinic of enrolment**  MTRH  Chulaimbo + Matayos^a^  Kitale  Huruma  UGDH | Ref.  1.37 (0.63 - 2.99)  2.40 (1.29 - 4.45)  0.56 (0.19 - 1.64)  1.58 (0.69 - 3.61) | 0.43  0.01  0.29  0.28 |

aOR, adjusted Odds Ratio; CI, Confidence Interval; MTRH, Moi Teaching and Referral Hospital; UGDH, Uasin Gishu District Hospital.

^a^ The Chulaimbo and Matayos clinics were combined in multivariate analyses due to few participants at the Matayos clinic. The clinics were similar in terms of demography, geography, and HIV prevalence.

The analysis was also adjusted for the number of text messages received in the study.
